# Identification of a novel cell culture adaptation site on the capsid of foot-and-mouth disease virus

**DOI:** 10.1099/jgv.0.000222

**Published:** 2015-09

**Authors:** Kyle Chamberlain, Veronica L. Fowler, Paul V. Barnett, Sarah Gold, Jemma Wadsworth, Nick J. Knowles, Terry Jackson

**Affiliations:** The Pirbright Institute, Ash Road, Pirbright, Woking, Surrey GU24 0NF, UK

## Abstract

Vaccination remains the most effective tool for control of foot-and-mouth disease both in endemic countries and as an emergency preparedness for new outbreaks. Foot-and-mouth disease vaccines are chemically inactivated virus preparations and the production of new vaccines is critically dependent upon cell culture adaptation of field viruses, which can prove problematic. A major driver of cell culture adaptation is receptor availability. Field isolates of foot-and-mouth disease virus (FMDV) use RGD-dependent integrins as receptors, whereas cell culture adaptation often selects for variants with altered receptor preferences. Previously, two independent sites on the capsid have been identified where mutations are associated with improved cell culture growth. One is a shallow depression formed by the three major structural proteins (VP1–VP3) where mutations create a heparan sulphate (HS)-binding site (the canonical HS-binding site). The other involves residues of VP1 and is located at the fivefold symmetry axis. For some viruses, changes at this site result in HS binding; for others, the receptors are unknown. Here, we report the identification of a novel site on VP2 where mutations resulted in an expanded cell tropism of a vaccine variant of A/IRN/87 (called A − ). Furthermore, we show that introducing the same mutations into a different type A field virus (A/TUR/2/2006) resulted in the same expanded cell culture tropism as the A/IRN/87 A −  vaccine variant. These observations add to the evidence for multiple cell attachment mechanisms for FMDV and may be useful for vaccine manufacture when cell culture adaptation proves difficult.

## Introduction

Foot-and-mouth disease is widespread throughout the world, affects livestock such as cattle, sheep, goats and pigs, and causes significant economic losses due to reduced productivity and trade restrictions on animals and animal products that are imposed on affected countries (Knight-Jones & Rushton, 2013). The aetiological agent, *Foot-and-mouth disease virus* (FMDV), is the type species of the genus *Aphthovirus* of the family *Picornaviridae*, a family of non-enveloped, single-stranded positive-sense RNA viruses. The viral capsid is formed by 60 copies each of four structural proteins (VP1–VP4) arranged in icosahedral symmetry. VP1–VP3 form the outer capsid surfaces, whilst VP4 is located within the capsid along with the genomic viral RNA (vRNA) (Jackson *et al.*, 2003). The viral non-structural proteins modify the cellular environment to favour virus production and, along with the 5′ and 3′ UTRs of the viral genome, direct replication of the vRNA (Mason *et al.*, 2003).

FMDV exists as seven serotypes [O, A, C, Asia-1, and Southern African Territories (SAT) SAT-1, SAT-2 and SAT-3], which each contain multiple and constantly evolving subserotype strains (Domingo *et al.*, 2003). A lack of immunological cross-reactivity between serotypes, and between some strains within a serotype, greatly complicates efforts to control foot-and-mouth disease by vaccination (Rodriguez & Gay, 2011). Accordingly, the most effective vaccines closely match the outbreak virus, which can necessitate the periodic development of new vaccine strains (Paton *et al.*, 2009). The current vaccines are chemically inactivated virus preparations grown in large-scale cell culture; hence, the production of new vaccines is critically dependent upon adaptation of field viruses for growth in cell culture, which can prove problematic for some viruses.

Field isolates of FMDV use RGD-dependent integrins as receptors to initiate infection (Berinstein *et al.*, 1995; Berryman *et al.*, 2005; Duque & Baxt, 2003; Jackson *et al.*, 2000, 2002, 2004; O'Donnell *et al.*, 2005). Integrin binding is mediated by an extended motif that includes an RGD tripeptide located on the G-H loop (the integrin-binding loop) of VP1 (Burman *et al.*, 2006; DiCara *et al.*, 2008). This loop is predicted to exist in two predominant conformations, ‘up’ and ‘down’, and when in the down position it adopts an ordered conformation and lies over VP2 (Logan *et al.*, 1993; Parry *et al.*, 1990), which results in some rearrangement of VP2 (residues 130–132) and refolding of VP3 (residues 172–180) away from the surface of VP2. A major driver of cell culture adaptation is receptor availability and cell culture adaptation often results in the selection of FMDV variants with altered receptor preferences that are no longer dependent on integrins for infection (Baranowski *et al.*, 2000; Jackson *et al.*, 1996; Sa-Carvalho *et al.*, 1997). As a consequence, cell culture adapted viruses have an expanded tropism for cultured cells and the ability to infect cells (e.g. CHO) that lack expression of the known FMDV integrin receptors. Remarkably, cell culture adaptation often arises from only a small number of residue changes that can occur at multiple sites on the capsid surface. Often these changes result in a net gain in positive charge and the ability to use negatively charged molecules [such as glycosaminoglycans, e.g. heparan sulphate (HS)] as receptors. An HS-binding site was originally identified using FMDV O1BFS 1860 and the structure of the virus in a complex with heparin (a structural mimic of HS) shows that the binding site is formed by a shallow depression that involves all three outer capsid proteins (VP1–VP3) (Fry *et al.*, 1999). The most important change that allows HS binding is at VP3 56 which is often H in field viruses and changes to R on cell culture adaptation. The R at VP3 56 occupies a central position in the depression and makes a major contribution to binding through ionic interactions with two sulfate groups on heparin. The other main contact residues are R 135 of VP2, which plays a subsidiary role in interacting via water molecules with one of the heparin disaccharides and an H that is located close to the C terminus of VP1 (residue 195). A similar HS-binding site was subsequently identified for FMDV A10/Argentina/61 (A1061) (Fry *et al.*, 2005), which included the key R residues at VP3 56 and VP2 135, suggesting a structurally conserved mechanism for cell culture adaptation. However, changes at a second site (the fivefold symmetry axis of the capsid) are now known to also result in cell culture adaptation. These changes occur at either of adjacent sites on VP1 (83–85, type O and SAT2; 109–110, types C and A; 110–112, SAT1) and require the introduction of positively charged residues (Berryman *et al.*, 2013; Escarmís *et al.*, 1998; Maree *et al.*, 2010, 2011; Zhao *et al.*, 2003). For some viruses, changes at the fivefold axes result in HS binding; for others, the receptors are not known, suggesting the existence of non-integrin, non-HS receptors for FMDV. Furthermore, for some viruses, mutations at this location appear to enhance integrin use and allow viruses with KGE in place of the normal integrin-binding RGD motif to use α_v_β_6_ receptors (Berryman *et al.*, 2013; Zhao *et al.*, 2003).

Previously, we described a novel variant of FMDV A/IRN/87 (Fowler *et al.*, 2010) with a major deletion (residues 141–153) within the G-H loop of VP1 that included the integrin-binding RGD motif (A/IRN/87/A − ; referred to here as A − ). This virus was isolated from a vaccine that also contained a related variant of A/IRN/2/87 that had an intact G-H loop (referred to here as A+). Despite having a major deletion at the integrin-binding site, the A −  virus was shown to grow normally in BHK-21 cells (Fowler *et al.*, 2011) and retained infectivity for cattle (Fowler *et al.*, 2014). These viruses (A+ and A − ) lack both the key HS contact residues identified in O1BFS 1860 and A1061 (a canonical HS-binding site), and the mutations at the fivefold symmetry axis associated with cell culture adaptation. Here, we show that both the A+ and A −  viruses are infectious for CHO cells (which lack the known integrin receptors of FMDV) and HS-deficient CHO-677 cells, suggesting they use novel receptors that are neither HS nor integrin. Using an infectious clone with the A −  virus capsid, we identified the amino acid changes that account for the expanded cell tropism of the A+ and A −  viruses, and showed that the same changes could be made in A/TUR/2/2006, resulting in a virus that could also infect CHO and HS-deficient CHO-677 cells. Our results add to the evidence for the existence of novel cell attachment mechanisms for FMDV and will aid the design of recombinant vaccine viruses when culture adaptation of field viruses proves intractable.

## Results

### Characterization of recombinant virus O1K/A− (vO1K/A−)

To enable site-directed mutagenesis of the A −  virus capsid, we constructed an infectious-copy plasmid (pO1K/A − ) using an approach known as ‘capsid switching’. This involved replacing the coding region for the capsid proteins VP1, VP2 and VP3 of an existing infectious clone of FMDV [O1Kaufbeuren (O1K)] with the corresponding region of the A −  virus. This approach has been used previously by us and others to produce viable, chimeric viruses that have capsids derived from FMDV field isolates paired with the non-structural proteins of a heterologous virus (Baranowski *et al.*, 2000; Berryman *et al.*, 2013; Maree *et al.*, 2010; Zhao *et al.*, 2003). The predicted amino acid sequence for the cloned PCR product for the A −  capsid (that was used to make pO1K/A − ) showed two residue differences in VP3 to the consensus sequence that was obtained previously by direct sequencing of PCR products (T136 and L210 in pO1K/A −  are A136 and F210 in the consensus sequence; Fig. S1, available in the online Supplementary Material). A chimeric virus (vO1K/A − ) was recovered by transfection using synthetic vRNA made from pO1K/A −  and subsequent passage using BHK cells. Complete cytopathic effect (CPE) was seen at the first passage (BHKp1). Direct sequencing of reverse transcription (RT)-PCR products for the entire capsid region of the recovered virus at the second and fourth passage (BHKp2 and BHKp4) confirmed the presence of VP4 of O1K, and VP1, VP2 and VP3 of A − .

**Fig. 1.**
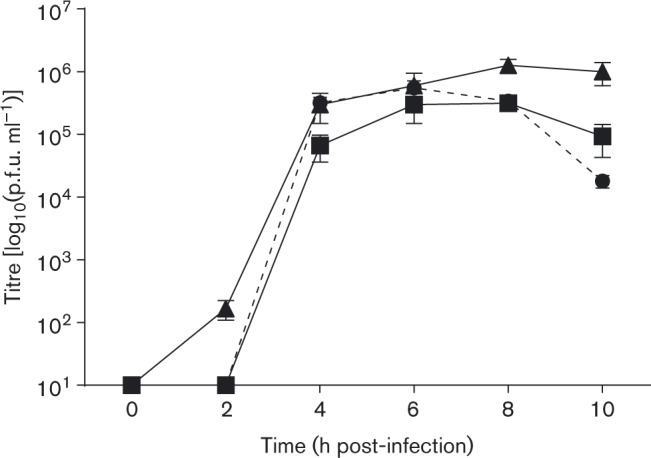
BHK cells in six-well plates were infected with A/IRN/87/A −  (▪), vO1K/A −  (▴) or O1K (derived from pT7S3; see Methods) (broken line and •) at m.o.i. 0.4 p.f.u. per cell for 0.5 h. Remaining extracellular virus was inactivated by a low-pH wash and infection continued in fresh media at 37 °C. At the indicated times post-infection, virus in cell supernatants was titrated on BHK cells. Data show mean ± sd for triplicate wells.

Next, we compared the cell culture tropism of the A/IRN/2/87 field isolate, the A+ and A −  viruses, and vO1K/A −  (BHKp2). All four viruses were infectious for BHK cells. The end-point titres for A+, A −  and vO1K/A −  were higher than for the field isolate. Furthermore, A+, A −  and vO1K/A − , but not the field isolate, were infectious for CHO and HS-deficient CHO cells (CHO-677) (Table 1). One-step growth curve analysis (Fig. 1) showed that vO1K/A −  replicated in BHK cells with similar kinetics as the A −  virus and the O1K virus from which the non-structural proteins of vO1K/A −  were derived. These results confirmed that the A+ and A −  viruses had a wider cell tropism than the field isolate and infected cells independently of the known integrin receptors of FMDV (by infection of CHO cells) and HS (by infection of CHO-677 cells). Furthermore, these results showed that vO1K/A −  had the same cell culture preference of the A −  virus and showed normal replication kinetics in BHK cells.

**Table 1.** The A/IRN/2/87 field isolate, A+ (A/IRN/87/A+) and A −  (A/IRN/87/A − ) viruses, and vO1K/A −  were titrated on the indicated cell lines vO1K/A − /VP2^78–80^AAR caused CPE on all three cell lines tested. ( − ) No CPE; (+) CPE, but the virus titre was not determined; pd, partial deletion (residues 141–153) within the G-H loop of VP1; nr, virus could not be recovered; nd, not done.

Virus	VP2		VP1	Infectivity		
	78–80	130–131	G-H loop	BHK-21	CHO	CHO-677
A/IRN/2/87 field isolate	LEK	KE	Intact	2.9 × 10^5^	( − )	( − )
A/IRN/87/A+	SAR	KK	Intact	3.0 × 10^6^	2.0 × 10^5^	(+)
A/IRN/87/A −	SAR	EK	pd	3.5 × 10^7^	2.5 × 10^5^	(+)
vO1K/A −	SAR	EK	pd	2.5 × 10^6^	1.5 × 10^5^	(+)
vO1K/A − /VP2^78–80^AAA	AAA	EK	pd	nr		
vO1K/A − /VP2^78–80^SAA	SAA	EK	pd	nr		
vO1K/A − /VP2^78–80^AAR	AAR	EK	pd	(+)	(+)	(+)
vO1K/A − /VP2^130–131^AA	SAR	AA	pd	nr		
vO1K/A − /VP2^130–131^KE	SAR	KE	pd	(+)*	nd	nd

* Recovered virus had KK at VP2 130–131.

### Identification of capsid residues with the potential to determine cell tropism

**Fig. 2.**
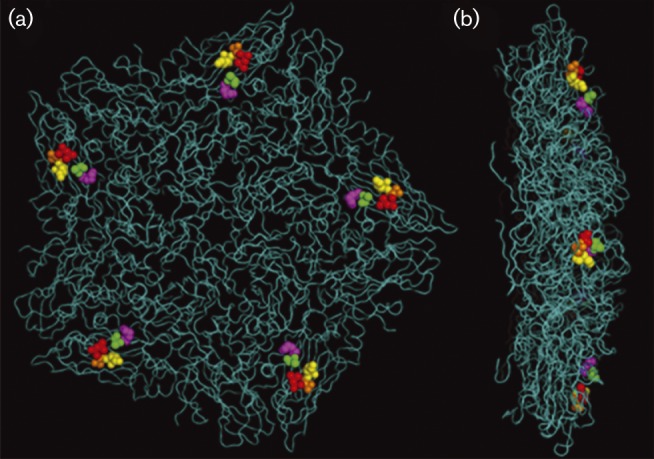
Location of VP2 residues 78 (red), 79 (orange) and 80 (yellow), and 130 (magenta) and 131 (green) using the crystallographic structure of a pentamer of FMDV A1061. In vO1K/A − , these residues are SAR and EK respectively. (Note that in A1061, residues 78–80 are LEK and 130–131 are KE.) (a) Outer surface of a pentamer. (b) A pentamer from a side-on view.

To identify residues that could account for the expanded cell culture tropism of the A −  virus, we compared the capsid sequence of A − , A+ and vO1K/A −  with the field isolate (Fig. S1). The changes associated with cell culture tropism would be expected to be common to the A+ and A −  viruses and vO1K/A −  as they are all infectious for CHO and HS-deficient CHO-677 cells (Table 1). The VP3 residues that differed between vO1K/A −  and the consensus sequence of A −  were discounted as they were not present in A+. Similarly, two other residues (VP1 138 and 155) that were different in A −  (and vO1K/A − ; A138 and A155) compared with the field isolate (V138 and K155) were discounted as they were not present in the A+ virus. In addition, three other residues in VP1 (141, 142 and 148) that were different between A+ and the field isolate were discounted as they are deleted in A − . There were nine other residue differences from the field isolate that were common to vO1K/A −  and the A −  and A+ viruses (Fig. S1). These were mapped onto the crystal structure of a related type A FMDV (A1061) (Fry *et al.*, 2005); this showed that four (VP2 88, 110, 193; VP3 196) were located at the inter-pentamer interface and one was buried within VP3 (VP3 85) where they were unlikely to directly influence receptor specificity and cell tropism (Fig. S2). The other four residues were in VP2 and clustered together at the capsid surface (Fig. 2). These residue were at 78–80, which were LEK in the field isolate and SAR in A+, A −  and vO1K/A − , and 131 which was E in the field isolate and K in A+, A −  and vO1K/A − . A further residue difference was seen at VP2 130, which was K in the field isolate and A+, but E in A −  and vO1K/A − . These observations suggested that the SAR residues at VP2 78–80 and EK at 130–131 (or KK in A+) could influence cell tropism of the A −  virus and were selected for further study.

### Investigating the requirement for VP2 78–80 SAR and 130–131 EK for A/IRN/87/A− infection

Initially, we investigated the role of the VP2, 78–80 SAR whilst leaving the EK motif intact. Mutations were made in pO1K/A −  that changed the SAR to SAA, AAA or AAR. Infectious virus could not be recovered (using BHK cells) from the clones containing SAA (pO1K/A − /VP2^78–80^SAA) or AAA (pO1K/A − /VP2^78–80^AAA) (Table 1). In contrast, virus was recovered from pO1K/A −  (used as a positive control for virus recovery) and for the mutant virus with AAR (vO1K/A − /VP2^78–80^AAR) (Table 1). Direct sequencing of PCR products for the capsids of the AAR viruses recovered at BHKp2 and BHKp4 confirmed the presence of the AAR motif, and that no other unwanted mutations had been introduced. The above experiments were carried out three times for each mutant (AAA, SAA and AAR) using at least two different vRNA preparations with the same outcomes. These results showed that the R at VP2 80 was required for infectivity of vO1K/A − . To determine if the AAR virus retained the expanded cell tropism of A − , we infected CHO and HS-deficient CHO-677 cells (Table 1). This analysis showed that vO1K/A − /VP2^78–80^AAR (BHKp4) was infectious for both cell types, thereby confirming that the AAR virus had the same tropism for CHO and HS-deficient CHO-677 cells as vO1K/A − .

The same approach was used to investigate the role of the EK motif at VP2 130–131. Mutations were made that introduced KE (pO1K/A − /VP2^130–131^KE) (i.e. the sequence of the field isolate) or AA (pO1K/A − /VP2^130–131^AA) in place of the EK sequence, whilst leaving the SAR at VP2 78–80 intact. Infectious virus could not be recovered from the AA clone despite repeated attempts using different batches of vRNA (Table 1). In contrast, infectious virus (vO1K/A − /VP2^130–131^KE) was recovered from the KE clone. The capsid sequences of the recovered virus (BHKp3) showed a single residue difference to the input RNA at VP2 131 (E to K), thereby creating the same sequence as the A+ virus (i.e. KK at VP2 130–131). These results showed that the E to K change at VP2 131 (seen in A+ and A − ) was required for infectivity and that either E or K could occupy VP2 130.

### SAR and EK motifs can be successfully transferred to other viruses

Taken together, the above observations indicated that the VP2 SAR (78–80) and EK (or KK for A+) motifs accounted for the expanded cell tropism of the A −  virus. Previously, we described a recombinant virus derived from an infectious-copy plasmid that was generated by ‘capsid switching’ using the O1K backbone and the capsid (VP1, VP2 and VP3) of the A/TUR/2/2006 field isolate (Berryman *et al.*, 2013) (from here called vO1K/A/TUR). As with the A/IRN/2/87 field isolate, vO1K/A/TUR had LEK and KE at VP2 78–80 and 130–131, respectively (Table 2), and was infectious for BHK but not CHO cells (Berryman *et al.*, 2013). Here, we investigated if the SAR and EK motifs could be introduced into vO1K/A/TUR, and if these changes resulted in the same expanded cell tropism as the A −  virus.

**Table 2.** The indicated viruses were titrated on BHK-21, CHO and CHO-677 cells (+) CPE, but the virus titre was not determined; ( − ) no CPE; nr, virus could not be recovered; nd, not done.

Virus	VP2		VP1		Infectivity		
	78–80	130–131	143–145	110	BHK-21	CHO	CHO-677
vO1K/A/TUR	LEK	KE	RGD	Q	(+)*	( − )*	( − )*
vO1K/A/TUR/VP1^110^A	LEK	KE	RGD	A	(+)	nd	nd
vO1K/A/TUR/VP1^110^A/VP2^78–80^SAR	SAR	KE	RGD	A	(+)	nd	nd
vO1K/A/TUR/VP1^110^A/VP2^78–80^SAR/VP1^143–145^KGA	SAR	KE	KGE	A	nr		
vO1K/A/TUR/VP1^110^A/VP1^143–145^KGA	LEK	KE	KGE	A	nr		
vO1K/A/TUR/VP1^110^A/VP1^143–145^KGA/VP2^130–131^KK	LEK	KK	KGE	A	nr		
vO1K/A/TUR/VP1^110^A/VP1^143–145^KGA/VP2^130–131^EK	LEK	EK	KGE	A	nr		
vO1K/A/TUR/VP1^110^A/VP1^143–145^	SAR	KK	KGE	A	2.0 × 10^6^	1.5 × 10^6^	nd
KGA/VP2^78–80^SAR/VP2^130–131^KK							
vO1K/A/TUR/VP1^110^A/VP1^143–145^	SAR	EK	KGE	A	4.7 × 10^5^	1.5 × 10^5^	(+)
KGA/VP2^78–80^SAR/VP2^130–131^EK							

* See Berryman *et al.* (2013).

Using pO1K/A/TUR as the backbone, we first investigated if changing the LEK at VP2 78–80 to SAR increased the cell tropism whilst leaving the KE at VP2 130–131 intact. Previously, we showed that during BHK cell passage, A/TUR/2/2006 (and vO1K/A/TUR) rapidly acquired a Q to K change at VP1 110 that allowed for infection of CHO cells (Berryman *et al.*, 2013). To reduce this possibility during our experiments we first changed the Q (codon: CAG) at VP1 110 to A (codon: GCA). This created a virus (vO1K/A/TUR/VP1^110^A) that required at least two nucleotide changes to introduce K at this site. Infectious virus (vO1K/A/TUR/VP1^110^A) was recovered (Table 2) and sequencing (BHKp2) of the capsid-encoding region confirmed the presence of the A at VP1 110 and no other unwanted mutations. Using pO1K/A/TUR/VP1^110^A as the template, we then mutated the VP2 78–80 LEK to SAR (to produce pO1K/A/TUR/VP1^110^A/VP2^78–80^SAR). Again, infectious virus was recovered (Table 2) and sequencing of the capsid-encoding region (BHKp2) confirmed the presence of the SAR motif and the A at VP1 110. This showed that the introduction of both A at VP1 110 and SAR at VP2 78–80 did not abrogate infectivity of vO1K/A/TUR.

In the above experiment, the resulting virus (vO1K/A/TUR/VP1^110^A/VP2^78–80^SAR) retained the potential to use integrin receptors to initiate infection due to the presence of an intact VP1 G-H loop (i.e. the integrin-binding RGD motif at VP1 143–145). Therefore, to prevent infection mediated by integrins we also mutated the VP1 G-H loop RGD sequence to KGA to produce pO1K/A/TUR/VP1^110^A/VP2^78–80^SAR/VP1^143–145^KGA. Sequencing of this plasmid confirmed the presence of the desired changes and that no other unwanted mutations had been introduced. However, repeated attempts to rescue virus (using BHK cells) were unsuccessful (Table 2). This showed that the introduction of SAR alone was insufficient to rescue infectivity of the virus with KGA in place of the RGD.

Next, we investigated the residues at VP2 130–131. Using pO1K/A/TUR as the template, we mutated both the Q at VP1 110 to A and the RGD at VP1 143–145 to KGA to produce pO1K/A/TUR/VP1^110^A/VP1^143–145^KGA. As expected, we were unable to recover virus using BHK cells and RNA from this plasmid (Table 2). Using pO1K/A/TUR/VP1^110^A/VP1^143–145^KGA as the template, we then changed the KE at VP2 130–131 to either KK (as in A+) or EK (as in A+ and vO1K/A − ) to produce plasmids pO1K/A/TUR/VP1^110^A/VP1^143–145^KGA/VP2^130–131^KK and pO1K/A/TUR/VP1^110^A/VP1^143–145^KGA/VP2^130–131^EK. Repeated attempts (using BHK cells) to recover virus also proved unsuccessful (Table 2), showing that the introduction of KK or EK at VP2 130–131 alone could not rescue infectivity of a virus with KGA in place of the RGD.

Finally, we used pO1K/A/TUR/VP1^110^A/VP1^143–145^KGA/VP2^130–131^KK and pO1K/A/TUR/VP1^110^A/VP1^143–145^KGA/VP2^130–131^EK as templates to introduce the SAR at VP2 78–80. This created infectious-copy plasmids with A at VP1 110, KGA at VP1 143–145 in place of the RGD, SAR at VP2 78–80 and either KK (as in A+) or EK (as in A −  and vO1K/A − ) at VP2 130–131. Infectious virus was readily recovered (Table 2) and sequencing of the capsid encoding regions at BHKp2 showed that for both viruses the sequence of the input RNA was retained. Table 2 shows that both viruses (vO1K/A/TUR/VP1^110^A/VP1^143–145^KGA/VP2^78–80^SAR/VP2^130–131^KK and vO1K/A/TUR/VP1^110^A/VP1^143–145^KGA/VP2^78–80^SAR/VP2^130–131^EK) had the same cell tropism as the A+ and A −  viruses, and could infect both CHO and HS-deficient CHO-677 cells. These results showed that the SAR and EK (or KK) sequences were required in combination to restore infectivity in the absence of a functional integrin-binding loop. Taken together, our results supported the conclusion that the SAR and KK/EK motifs at VP2 78–80 and 130–131 were the critical residues responsible for the expanded cell tropism of the A+ and A −  viruses, and that these residues could be transferred to a second type A virus, resulting in the same expanded cell tropism.

## Discussion

Foot-and-mouth disease is one of the most prevalent epizootic diseases and affects huge numbers of animals, including domesticated livestock (e.g. cattle, sheep, goats and pigs) and >70 wildlife species (Arzt *et al.*, 2011). Foot-and-mouth disease remains endemic in many countries in Africa and Asia, where it causes enormous economic problems due to production and trade losses (Knight-Jones & Rushton, 2013). In addition, sporadic outbreaks frequently occur in countries that are normally free from disease, such as those in the UK in 2001 and 2007, and more recently in Bulgaria, Japan, South Korea, Russia and extensively in China. Foot-and-mouth disease is difficult and expensive to control as the causative virus (FMDV) is highly contagious and spreads rapidly through susceptible animals. Vaccination is an important tool for controlling foot-and-mouth disease and it is estimated that 2.6 billion doses of vaccine are administered annually (Knight-Jones & Rushton, 2013). Control by vaccination is complicated by many factors, including the existence of seven, constantly evolving, antigenically distinct serotypes. This results in the periodic emergence of novel variants for which the current vaccines are a poor match and the consequent need to develop new vaccine strains from the field. Field viruses grow poorly in cell culture and, therefore, the production of new vaccines requires a period of cell culture adaptation. For some viruses, (e.g. type O viruses) cell culture adaptation can occur readily; for others, it can be time consuming or in some cases unfruitful. Thus, understanding the mechanisms of cell culture adaptation could lead to the design of recombinant FMDVs with ‘in-built’, improved cell culture growth which could reduce the time taken for the development of new vaccines from field-isolated viruses.

**Fig. 3.**
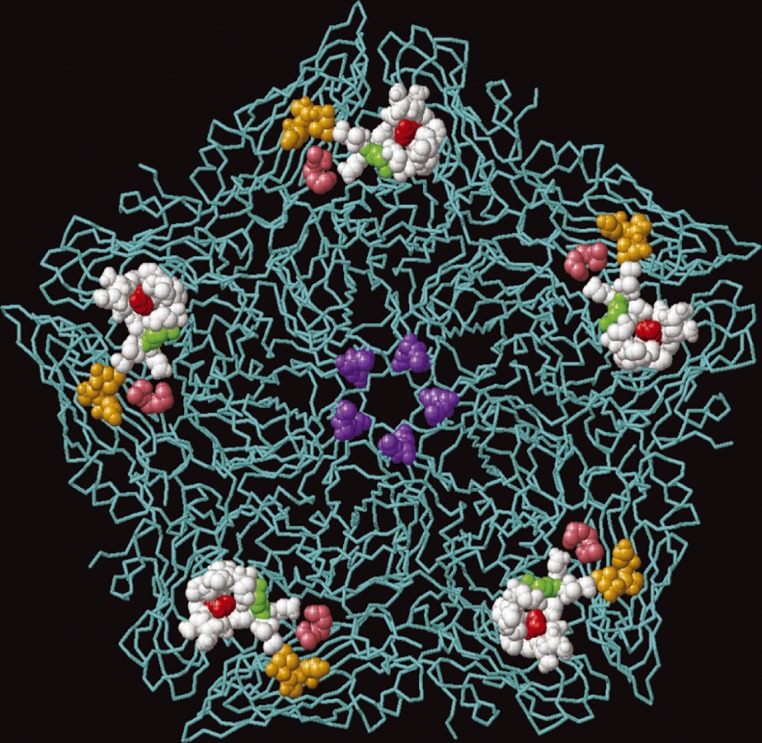
Relative positions of the known sites on the outer capsid where amino acid changes are associated with an expanded cell tropism of type A FMDV mapped onto a pentamer of FMDV A1061. The residues (VP1, 109 and 110) at the fivefold symmetry axis associated with an expanded cell tropism of the A/TUR/2/2006 field isolate are shown in purple. The residues (VP2 134–138; VP3 55–60 and VP1 193–195) that form the canonical HS-binding site (identified on FMDV A1061) are coloured white. The key HS contact residues are shown in red (VP3 56) and green (VP3 135). The positions occupied by SAR at VP2 78–80 and EK at VP2 130–131 of A/IRN/87/A −  are coloured orange and pink, respectively.

Studies of FMDV cell culture adaptation have shown that it involves changes in the capsid proteins and the selection of variants with altered receptor preferences that are no longer dependent on integrins to initiate infection. Remarkably, these changes can occur at independent sites, and often involve only a small number of residue changes and a net gain of positive charge on the outer capsid surfaces. Here, we have identified a novel site on the capsid of a variant of A/IRN/2/87 (called A/IRN/87/A − ) where changes result in cell culture adaptation. This virus is unique among FMDV as it retains infectivity for cattle, despite having a major deletion in the G-H loop of VP1 that results in the loss of the integrin-binding RGD (Fowler *et al.*, 2010, 2014). The cell culture changes we identified occur at a site that is distinct from the canonical HS-binding site and the fivefold symmetry axis (Fig. 3), and is formed exclusively by VP2 residues 78–80 (SAR) and 130–131 [EK (A − ) or KK (A+)]. The most likely explanation to account for our observations is that the SAR and EK (or KK) motifs associate to form a receptor binding site; a conclusion supported by their close proximity on the outer capsid surface. This conclusion is also supported by showing that the SAR and EK motifs were both required to rescue infectivity of a second type A virus (vO1K/A/TUR) with a non-functional KGA in place of the normal integrin-binding RGD motif. In agreement with other reported cell culture mutations, the changes associated with cell culture adaptation of A/IRN/2/87 (78–80 LEK to SAR and 130–131 KE to EK) results in a net gain of positive charge; in addition, the substitution of KE by EK at VP1 130–131 places a positively charged residue (the K at 131) closer to the positively charged R at VP2 80, thereby potentially increasing the local positive charge at this site (Fig. 2), which could allow for stronger interactions with negatively charged moieties (e.g. glycosaminoglycans) at the cell surface. However, the ability to infect CHO-677 cells suggests that the A −  virus is not dependent on HS receptors for infection. Furthermore, cell-culture-adapted viruses that bind HS have been shown to be attenuated for cattle (Sa-Carvalho *et al.*, 1997); however, despite being cell culture adapted and lacking an integrin-binding site, the A −  virus was shown previously to be infectious for cattle, although disease was mild compared with the field isolate (Fowler *et al.*, 2014). Together, these observations support the conclusion that the A −  virus does not need HS for infection and the identity of the receptor(s) used is currently unknown.

Our results suggest that the R at VP2 80 and the K at VP2 131 are the most important residues for cell culture adaptation of the A −  virus. We have interrogated the available sequence data, which show that LEK is predominantly found at VP2 78–80 (as in the A/IRN/2/87 field isolate) in type A viruses and that the K at position 80 appears to be strongly conserved. This analysis also showed that the SAR residues at VP2 78–80 are unique to the A+ and A −  viruses, and have not been seen before in any FMDV regardless of serotype. The KE (as in the A/IRN/2/87 field isolate) at VP2 130–131 is also strongly conserved in type A FMDV, although some viruses have KD at this site. In contrast to the LEK-to-SAR change, the E-to-K change at VP2 131 has been reported previously in a type A vaccine strain (FMDV A/IND/40/2000) (Biswal *et al.*, 2015). This virus had enhanced infectivity for BHK cells and was shown to bind heparin, but the requirement for HS receptors was not investigated. The A/IND/40/2000 virus has LEK at VP2 78–80, which suggests that, under certain circumstances, the presence of K at 131 can improve cell culture growth in the absence of an SAR motif. However, unlike the A −  virus, A/IND/40/2000 has an intact VP1 G-H loop and retains the potential to use integrin receptors; thus, the possibility that the presence of a K at VP2 131 leads to enhanced integrin-mediated infection cannot be excluded as proposed for FMDV with cell culture adaptations at the fivefold symmetry axis (Berryman *et al.*, 2013; Zhao *et al.*, 2003).

In conclusion, we have identified capsid residues on VP2 (78–80 and 130–131) where amino acid changes account for the cell culture adaptation and extended cell tropism of a variant of FMDV, A/IRN/2/87. Importantly, we have shown that the same residue changes can be transferred to a second type A virus, resulting in the same extended cell tropism. Our results add to the evidence for multiple cell attachment mechanisms for FMDV and improve our understanding of FMDV cell culture adaptation. These observations may be useful for vaccine manufacture when cell culture adaptation of FMDV proves difficult.

## Methods

### Cells

Baby hamster kidney (BHK-21) cells were cultured in Dulbecco's modified Eagle's medium, and Chinese hamster ovary (CHO) and HS-deficient CHO pgsD-677 cells (CHO-677) in Hams F-12, each supplemented with 10 % FBS, 20 mM glutamine, penicillin (100 U ml^− 1^) and streptomycin (100 μg ml^− 1^).

### Viruses

The field isolate of FMDV A/IRN/2/87 was obtained from the Foot-and-Mouth Disease the World Reference Laboratory for Foot-and-mouth Disease at Pirbright. The vaccine virus A/IRN/87/A+ and the VP1 G-H loop deleted variant A/IRN/87/A −  were isolated from a Middle Eastern, type A vaccine strain (A/IRN/87) after three rounds of plaque purification as described previously (Fowler *et al.*, 2010).

### Virus and plasmid sequencing

The methods to obtain the capsid-encoding sequences of A/IRN/2/87/A+ and A/IRN/2/87/A −  by direct sequencing of PCR products have been described previously (Fowler *et al.*, 2010). Here, we used the same methods and primers to obtain the capsid-encoding sequence of the A/IRN/2/87 field isolate except that TRIzol (Life Technologies) was used to extract RNA and virus was propagated using primary bovine thyroid cells.

All infectious-copy plasmids with and without mutations, and PCR products for the capsid-coding region of the recovered viruses, were sequenced using primers O1A-VP4 (5′-AACAACTACTACATGCAGC-3′) and O2B (5′-CTCCTGCATCTGGTTGATGG-3′) which anneal in the 2B and VP4 coding region of O1K, respectively, and the internal primers that were used to sequence A/IRN/2/87/A+ and A/IRN/2/87/A −  (Fowler *et al.*, 2010). Sequencing of TOPO clones used vector-specific primers. DNA templates were sequenced using a BigDye Terminator version 3.1 Cycle Sequencing kit (Applied Biosystems).

### Construction of pO1K/A− by capsid switching

Total RNA was extracted from A/IRN/2/87/A − infected BHK cell lysates using TRIzol (Invitrogen) as recommended by the manufacturer and resuspended in nuclease-free water. cDNA for the capsid-coding region was generated using a SuperScript III One-Step RT-PCR kit (Invitrogen) and primers 5′-CACTGCTAGCCGACAAAAAGACAGAGGAAACTA-3′, which introduces an *Nhe*I site (underlined) at the VP4/VP2 junction, and 5′-GAAGGGCCCAGGGTTGGATTCAACGTCT-3′, which introduces an *Apa*I site (underlined) at the 2A/2B junction. The resulting PCR product was gel-purified and ligated into pCR-Blunt II-TOPO and sequenced. The *Nhe*I/*Apa*I fragment, which includes the coding regions for VP2, VP3, VP1 and 2A of A/IRN/2/87/A − , was used to replace the corresponding coding sequences of plasmid pT7S3 (the O1K infectious-copy plasmid) to create pO1K/A − . The detailed methods describing this approach have been published previously (Bøtner *et al.*, 2011; Lohse *et al.*, 2012).

### Site-directed mutagenesis

All mutations were introduced using the QuikChange Lightning Site-Directed mutagenesis kit (Agilent Technologies) and the appropriate primer pairs listed in Table S1.

### Virus rescue

Plasmids were linearized by digestion with *Hpa*I. Synthetic, full-length vRNA was made using a T7 Ambion MEGAscript kit and subjected to DNase digestion using TurboDNase (Life Technolgies), and purified using a MEGAclear Transcription Clean-Up kit (Life Technolgies). RNA quality and concentration were determined by agarose gel electrophoresis and nanodrop, respectively. BHK cells were transfected with vRNA by electroporation as described previously (Bøtner *et al.*, 2011). At 24 h post-transfection, cell lysates were prepared by freeze/thawing overnight and clarified by centrifugation. For all transfections (including non-recoverable mutants), viruses in the cell lysates were passaged through BHK cells.

The sequence of the capsid-coding region of recovered viruses was obtained by direct sequencing of PCR products. Total RNA was extracted from 0.5 ml cell lysate using TRIzol (Invitrogen) as per the manufacturer's instructions and resuspended in 20 μl of RNase-free water. ssDNA was synthesized using an Invitrogen First-Strand cDNA Synthesis kit and primer O2B. PCR used KOD polymerase (Novagen) and primers O1A and O2B using cycling conditions: 95 °C for 2 min; 30 cycles of 95 °C for 20 s, 52 °C for 15 s and 70 °C for 40 s; a final incubation at 70 °C for 1 min. For each virus, a control PCR was carried out that used, as the template, a cDNA synthesis reaction in the absence of reverse transcriptase. The control PCRs were negative, confirming that PCR capsid products were derived from vRNA and not from contaminating plasmid DNA.

### Infections

Virus passage of rescued viruses. BHK cell monolayers (∼90 % confluent) in 25 cm^2^ flasks were infected with FMDV (1 ml lysate) in 5 ml virus growth medium (normal cell culture medium with 1 % FCS) for a maximum of 48 h. The cells were freeze/thawed, and the resulting lysate clarified by centrifugation and stored at − 80 °C.

Virus titrations. Cell (BHK, CHO and CHO-677) monolayers (∼90 % confluent) in 24-well plates were incubated with FMDV at room temperature for 15 min. Additional media was added to the wells without removing the virus inoculum and infection continued at 37 °C for 20 h. Virus in the cell supernatants was titrated on BHK cells using a standard plaque assay as described previously (Jackson *et al.*, 1996).

### One-step growth curve

BHK cell monolayers (∼90 % confluent) in 25 cm^2^ flasks were infected with FMDV at 0.5 p.f.u. per cell. Triplicate flasks were used for each virus. At 0.5 h post-infection, the virus inocula were removed and the cells incubated with low pH buffer (pH 5.2) for 2 min followed by washing with cell culture media to restore the pH to neutrality. At the indicated times, samples of cell supernatants were titrated on BHK cells as described above.

## Supplementary Data

166 Supplementary Data
