# Supplementary material for: Identification of a novel cell culture adaptation site on the capsid of foot-and-mouth disease virus
Source: J Gen Virol. 2015 Sep;96(Pt 9):2684–92. doi: 10.1099/jgv.0.000222 (PMC4635497; doi:10.1099/jgv.0.000222)
Supplement: Supplementary file 1 — Supplementary Data [file vir-96-09-2684-s001.pdf]

VP2

```
        10        20        30        40        50        60        70        80        90       100
....|....|....|....|....|....|....|....|....|....|....|....|....|....|....|....|....|....|....|....|....|....
DKKTEETTLLEDRILTTRNGHTTSTTQSSVGVTYGYSTGEDHVSGPNTSGLETRVVQAERFFKKHLFDWTPDKPFGHLEKLELPTEHTGVYGHLVESFAYMRNGWDVEV
-----------------------------------------------------------------------------SAR-------A---------------------
-----------------------------------------------------------------------------SAR-------A---------------------
-----------------------------------------------------------------------------SAR-------A---------------------

110       120       130       140       150       160       170       180       190       200       210
|....|....|....|....|....|....|....|....|....|....|....|....|....|....|....|....|....|....|....|....|....|...
SAVGNQFNGGCLLVAMVPEWKEFTQREKYQLTLFPHQFISPRTNMTAHITVPYLGVNRYDQYKKHKPWTLVVMVVSPLTTSSIAAGQIKVYANIAPTHVHVAGELPSKE
T--------------------K-------------------------------------------------------------S-------------------------
T-------------------EK-------------------------------------------------------------S-------------------------
T-------------------EK-------------------------------------------------------------S-------------------------
```

VP3

```
        10        20        30        40        50        60        70        80        90       100
....|....|....|....|....|....|....|....|....|....|....|....|....|....|....|....|....|....|....|....|....|....
GIVPVACSDGYGGLVTTDPKTADPVYGMVYNPPRTNYPGRFTNLLDVAEACPTLLCFENGKPYVETRTDDQRLLAKFDVSLAAKHMSNTYLAGIAQYYAQYSGTINLHF
------------------------------------------------------------------------------------P------------------------
------------------------------------------------------------------------------------P------------------------
------------------------------------------------------------------------------------P------------------------

110       120       130       140       150       160       170       180       190       200       210
|....|....|....|....|....|....|....|....|....|....|....|....|....|....|....|....|....|....|....|....|....|...

MFTGSTDSKARYMVAYVPPGVDTPPDAPERAAHCIHAEWDTGLNSKFTFSIPYMSAADYAYTASDVAETTNVQGWVCIYQITHGKAEQDTLVVSVSAGKDFELRLPIDP
--------------------------------------------------------------------------------------A----------------------
--------------------------------------------------------------------------------------A----------------------
--------------------------T-----------------------------------------------------------A-------------L--------

 220
.|.
RAQ
---
---
---
```

VP1

```
        10        20        30        40        50        60        70        80        90       100
....|....|....|....|....|....|....|....|....|....|....|....|....|....|....|....|....|....|....|....|....|....
TTATGESADPVTTTVENYGGETQVRRRQHTDVSFIMDRFVKINPVTPTHVIDLMQTHQHALVGALLRAATYYFSDLEIVVRHEGNLTWVPNGAPEAALSNTSNPTAYHK
-------------------------------------------------------------------------------------------------------------
-------------------------------------------------------------------------------------------------------------
-------------------------------------------------------------------------------------------------------------

110       120       130       140       150       160       170       180       190       200       210
|....|....|....|....|....|....|....|....|....|....|....|....|....|....|....|....|....|....|....|....|
EPFTRLALPYTAPHRVLATVYNGTNKYAATGARRGDLGSLAARVAAQLPSSFNFGAIRATTIHELLVRMRRAELYCPRPLLAMEVSAEGRHKQKIIAPAKQ
-------------------------------DS-----P--------------------------------------------------------------
----------------------------V--             -K-------------------------------------------------------
----------------------------V--             -K-------------------------------------------------------
```

Fig. S1. Capsid amino-acid sequence alignment

Shown is the amino acid sequence of VP2, VP3 and VP1. Upper sequence, the A/IRN/2/87 field isolate; Second sequence, the A+ virus; Third sequence, the consensus sequence of the A- virus; Lower sequence, vO1K/A-. A dash indicates identity with the field isolate. The region of VP1 G-H loop deleted in the A- virus and vO1K/A- are highlighted by the yellow bar. VP2 residues (78-80 and 131) are indicated by grey highlights. The K to E changes at VP2 130 seen in the A- virus and vO1K/A- are indicated by blue highlights. The T and L at VP3 136 and 210 respectively were only present in vO1K/A-. All other changes were at residues that are either buried within VP3 (VP3 85) or at the inter-pentamer interface (VP2: 88, 110, 193 and VP3 196) where they are unlikely to directly influence receptor specificity.

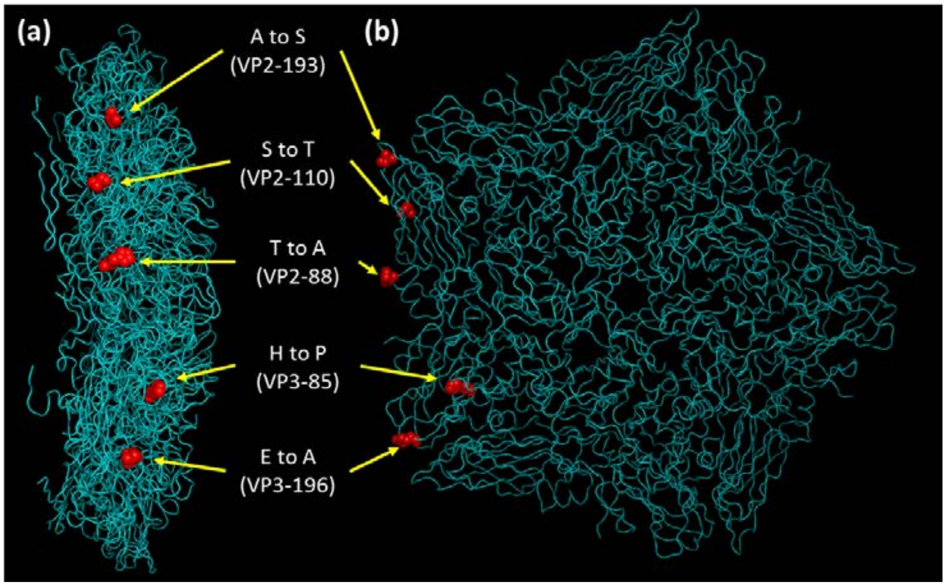

Fig. S2. Selected residues where the amino acids of the A/IRN/2/87 field isolate are different to the A+ and A- viruses. Residue positions are shown mapped onto the crystal structure of a pentamer of a related FMDV A_{10}61. In the virion, these residues are either located at the interface between adjacent pentamers (VP2 88, 110, 193 and VP3 196) or buried within VP3 (VP3-85). Panel (a) shows a side elevation and panel (b) a view of the outer capsid surface. Amino acids are given by the single letter code.

**Table S1. List of mutagenesis primer sequences**				
**vO1K/A-**	**Sequence 5'-3'**	**Site and mutation**		
F	AAGCCGTTTGGGCACGCAGCAGCGTTGGAACTCCCCACC	VP2 78-80	SAR → AAA
R	GGTGGGGAGTTCCAACGCTGCTGCGTGCCCAAACGGCTT		
F	AAGCCGTTTGGGCACTCAGCAGCGTTGGAACTCCCCACC		SAR → SAA
R	GGTGGGGAGTTCCAACGCTGCTGAGTGCCCAAACGGCTT		
F	AAGCCGTTTGGGCACGCAGCAAGATTGGAACTCCCCACC		SAR→ AAR
R	GGTGGGGAGTTCCAATCTTGCTGCGTGCCCAAACGGCTT		
F	GCCATGGTTCCTGAATGGGCAGCATTCACTCAGCGCGAG	VP2 130-131	EK → AA
R	CTCGCGCTGAGTGAATGCTGCCCATTCAGGAACCATGGC		
F	GCCATGGTTCCTGAATGGAAAGAGTTCACTCAGCGCGAG		EK → KE
R	CTCGCGCTGAGTGAACTCTTTCCATTCAGGAACCATGGC		
**vO1k/A/TUR**			
F	GGTAATGGCAGAAAAGGTGCGCTGGGGCCTC	VP1 143-145	RGD → KGA
R	GAGGCC-CAGCGCACCTTTTCTGCCATTACC		
F	GCCTACCACAAGGCACCATTTACGAGACTTGCG	VP1 110	Q → A
R	CGCAAGTCTCGTAAATGGTGCCTTGTGGTAGGC		
F	GCTTTTGGACACTCGGCAAGGCTGGAACTCCCC	VP2 78-80	LEK → SAR
R	GGGGAGTTCCAGCCTTGCCGAGTGTCCAAAAGC		
F	GCCATGGTACCTGAGTGGGAGAAATTTACCCCTCGTGAG	VP2 130-131	KE → EK
R	CTCACGAGGGGTAAATTTCTCCCACTCAGGTACCATGGC		
F	GCCATGGTACCTGAGTGGAAAAAATTTACCCCTCGTGAG		KE → KK
R	CTCACGAGGGGTAAATTTTTTCCACTCAGGTACCATGGC		
F: Forward primer; R: Reverse primer; Amino acids are shown using the single letter code			
